# Comparison of the Impacts of a Dynamic Exercise Program vs. a Mediterranean Diet on Serum Cytokine Concentrations in Women With Rheumatoid Arthritis. A Secondary Analysis of a Randomized Clinical Trial

**DOI:** 10.3389/fnut.2022.834824

**Published:** 2022-04-25

**Authors:** Mariel Lozada-Mellado, Luis Llorente, Andrea Hinojosa-Azaola, José M. García-Morales, Midori Ogata-Medel, Jorge Alcocer-Varela, Juan A. Pineda-Juárez, Lilia Castillo-Martínez

**Affiliations:** ^1^Clinical Nutrition Service, Instituto Nacional de Ciencias Médicas y Nutrición Salvador Zubirán, Mexico City, Mexico; ^2^Programa de Maestría y Doctorado en Ciencias Médicas, Odontológicas y de la Salud, Universidad Nacional Autónoma de México (UNAM), Mexico City, Mexico; ^3^Department of Immunology and Rheumatology, Instituto Nacional de Ciencias Médicas y Nutrición Salvador Zubirán, Mexico City, Mexico; ^4^Research Coordination, Centro Medico Nacional 20 de Noviembre, ISSSTE, Mexico City, Mexico

**Keywords:** exercise program, mediterranean diet, cytokines, rheumatoid arthritis, clinical trial

## Abstract

**Background:**

Rheumatoid arthritis (RA) is a disease characterized by a chronic inflammatory state. High pro-inflammatory cytokine levels are associated with disease activity. Exercise and the Mediterranean diet (MD) exert anti-inflammatory effects; however, their impacts on inflammation in RA patients remains unknown. This study aimed to compare the effects of six-months of dynamic exercise program (DEP) vs. MD on pro- and anti-inflammatory cytokine serum concentrations.

**Methods:**

Secondary analysis of a randomized clinical trial in which 90 women with RA were randomly assigned to the DEP (*n* = 30), MD (*n* = 30), or control group (*n* = 30). All patients received pharmacological treatment. Serum concentrations of pro-inflammatory (TNF-α, TNF-β, IL-1β, IL-6 pg/mL) and anti-inflammatory (IL-10, IL-Ra pg/mL) cytokines were measured at baseline and after 6 months using the Luminex technique.

**Results:**

After 6 months of follow-up, we found an improvement of the median percentages changes concentrations of TNF-α (DEP, −12.3; MD, −13.3; control, 73.2; *p* = 0.01), TNF-β (DEP, −67.4; MD, −54.9; control, 0; *p* = 0.04), and IL-6 (DEP, −19.9; MD, −37.7; control, 45.5; *p* = 0.04) in the DEP and MED groups in comparison with control group. IL-1Ra concentrations increased only in the MD group (13.8) compared to levels in the control group (−31.7), *p* = 0.04. There were no statistically significant differences between DEP and MD groups. Only *n* = 27 participants in the DEP group, *n* = 26 in the MD group, and *n* = 21 in the control group completed the follow-up.

**Conclusion:**

The DEP and the MD have potential effects in the concentrations of pro-inflammatory cytokines compared with those in a control group. Only the MD elevated the concentration of IL-Ra.

**Clinical Trial Registration:**

[ClinicalTrials.gov], identifier [NCT02900898].

## Introduction

Rheumatoid arthritis (RA) is a chronic disease of an autoimmune nature characterized by the destruction of the synovial membrane, inflammation, and joint pain (1). This disease has a high economic and social impact associated with increased mortality and premature disability (2). It is estimated to affect 1% of the adult population worldwide (3).

In the pathogenesis of RA, chronic inflammation of the synovial membrane occurs by the infiltration of T and B lymphocytes, endothelial cells, and macrophages that produce cytokines (low-molecular-weight proteins that mediate intercellular signal transmissions). They are usually classified as pro-inflammatory or anti-inflammatory. In RA, high cytokine concentrations have been associated with disease activity, structural joint damage, and poor prognosis. TNF-α, IL-1β, and IL-6 are among the most relevant pro-inflammatory cytokines in the pathogenesis of this disease (4–6). It has been observed that the concentrations of anti-inflammatory cytokines in RA patients are higher than those in healthy individuals (7); however, they do not efficiently counteract the inflammatory effects of their counterparts.

Pharmacological and non-pharmacological treatments aim to control symptoms and dampen the immune response (8). Non-pharmacological interventions such as diet and physical exercise have become relevant for autoimmune conditions due to their impacts on cardiac and endothelial functions and should be considered in the primary and secondary prevention of cardiovascular disease (9–11).

Previous studies have reported the benefits of exercise in RA patients (12, 13). When dynamic exercise programs (DEPs) are designed and implemented properly, they can improve cardiovascular health and physical function without increasing disease activity or causing joint damage (12). A DEP is defined as an exercise therapy of sufficient intensity, duration, and frequency to improve aerobic capacity and/or muscle strength and exert a positive effect on functional ability in RA patients (13).

On the other hand, the Mediterranean diet (MD) is characterized by the incorporation of large amounts of vegetables, fruits, cereals, and legumes. Olive oil is a significant source of fat in this diet, whereas dairy products are included mainly in the form of yogurt and cheese in moderate amounts. This diet is distinguished for being low in saturated fat but high in fiber, complex carbohydrates, monounsaturated fatty acids, and antioxidant vitamins (14). Several studies have evaluated the effect of the MD in healthy populations, concluding that it reduces the incidence of major cardiovascular events due to its anti-inflammatory properties (15–18).

We conducted a secondary analysis of a randomized clinical trial that included consecutive women with RA diagnosis. We evaluated the individual effect of a DEP or the MD on serum concentrations of pro-inflammatory cytokines (TNF-α, TNF-β, IL-1β, and IL-6) and anti-inflammatory cytokines (IL-10 and IL-1Ra) after 6 months of intervention. We hypothesized that in women with RA, 6 months of DEP or MD therapy would decrease the concentrations of TNF-α, TNF-β, IL-1β, and IL-6 compared to those in the control group.

## Materials and Methods

### Protocol Design and Registration

The present study is a secondary analysis of a clinical trial registered at ClinicalTrials.gov (ID: NCT02900898) that was undertaken from August 2016 to December 2019. Between June and July 2020, we performed this secondary analysis. The rationale for the trial design details and eligibility features, as well as the main results, have been published previously (19, 20).

### Participants

Briefly, in the clinical trial, women (aged ≥18 years) diagnosed with RA according to the 2010 criteria of the American College of Rheumatology/European League Against Rheumatism (1) and classified in functional classes I-III (21) were recruited consecutively between August 2016 and June 2019 at the Department of Immunology and Rheumatology of the Instituto Nacional de Ciencias Médicas y Nutrición Salvador Zubirán in Mexico City, Mexico. Patients were excluded if they had chronic heart failure, cancer, chronic kidney disease, HIV, or other autoimmune diseases in overlap with RA (e.g., Sjögren’s syndrome, systemic lupus erythematosus, fibromyalgia); if they performed structured exercise therapy; if they were on a specific diet or consumed food supplements within the previous 6 months; if they had a condition that did not allow them to perform physical activity (e.g., arthroplasty, functional class IV); or if they were receiving biological drugs for RA (in an attempt to homogenize groups, since only a few patients at our centre have access to biological drugs) (19, 20). While the clinical trial included a total of 144 women, this secondary analysis included 90 participants (62.5%) whose cytokine concentrations were measured before and after interventions.

All patients signed written informed consent prior to enrollment, and the Institutional Human Ethics and Research Committees approved the study (Ref: 1347).

Sample size was calculated using a multiple comparisons formula, which considered 95% accuracy and 80% power, according to the IL-6 mean change from previous studies (22), resulting in 30 patients per group.

Participants were randomly assigned using a sequence created by the website www.randomization.com to one of three groups: 1) DEP (*n* = 30), 2) MD (*n* = 30), and control group (*n* = 30). An external collaborator created the randomization sequence, and the researchers carried out the assignment process.

### Interventions

Full details of the MD dietary intervention have been previously published (21). It consisted of an individualized diet prescribed according to the estimated basal energy expenditure. The diet plan was based on the consumption of olive oil as the main dietary fat (23) (see Supplementary Material). All patients received individual verbal instructions from the dietitian and a nutritional handbook containing five menus. We used the Mediterranean Diet Score (MedScore) to evaluate adherence to the diet (24). All dietary data were analyzed using the ESHA Food Processor^®^ version 11.1. Patients randomized to the MD group received general physical activity recommendations.

The DEP intervention has been described previously (19) (see Supplementary Material). It consisted of a face-to-face program carried out at the physiotherapy gymnasium. Participants attended a total of 48 DEP sessions over the course of 24 weeks consisting of two 80–90-minute sessions per week. The intensity of the exercise was calculated using the Karvonen method (25). All sessions were conducted in groups with a maximum of 10 participants per group. Patients randomized to the DEP received general nutritional recommendations. Physical activity was evaluated before the study and 24 weeks after the intervention using a one-week self-report, paper and pencil, and an activity diary. “Exercise adherence” was defined as participation in at least 80% of the exercise sessions.

Participants assigned to the control group received verbal recommendations concerning physical activity guides and basic nutrition information (23, 26, 27). All patients, independent of intervention group, received conventional pharmacological therapy with disease-modifying antirheumatic drugs (DMARDs).

### Measurements

The primary outcome of this secondary analysis consisted of the differences in the cytokine concentration changes among the three groups.

### Cytokine Measurement

An independent investigator who was blinded to the group assignment of the patients performed the cytokine analysis. At baseline, 5-mL fasting blood samples were collected 1 week before the beginning of the interventions. Post-intervention measurement of cytokines was performed 72 h after the last exercise session in the DEP group and 6 months after the basal measurement in the MD and control groups. All samples were stored in gel serum separator tubes and centrifuged at 3,000 rpm for 15 min.

Cytokine quantification was performed using the Human Cytokine kit (Millipore, Billerica, MA, United States) by means of the Luminex^®^ technique according to the manufacturer’s instructions. Reading of plates was performed using Bio-Plex 200 equipment (Bio–Rad Laboratories, Hercules, CA, United States) with the Bio-Plex Manager Software. Cytokine concentrations were determined by evaluating the average fluorescence intensities in a calibration curve.

### Demographic and Clinical Characteristics

Two rheumatologists (blinded to group assignments) collected all clinical data related to RA, including symptoms, disease duration, associated comorbidities, and pharmacological treatment, and calculated disease activity score-28 (DAS28) values (28). Perception of pain was assessed using a visual analog scale (0–10), whereas disability was evaluated using the health assessment questionnaire disability index (HAQ-DI) (29). Acute phase reactants such as high-sensitivity C-reactive protein (CRP) and erythrocyte sedimentation rate (ESR) were determined using routinely automated analyzers.

### Statistical Analysis

The Shapiro–Wilk test was used to assess the normality of the variables. Data are reported as the mean ± standard deviation (SD) if normally distributed or as median and percentiles 25–75 (p25-p75) if otherwise, whereas categorical variables are presented as frequencies and percentages. The Wilcoxon test was used to evaluate the changes from baseline to final measurements within each group. For the post-intervention comparison of groups, percentage changes were calculated and analyzed with the Kruskal–Wallis test. *Post hoc* comparisons were performed using the Mann–Whitney U test with Bonferroni correction. All analyses were performed using SPSS Version 25 (SPSS Statistics for Windows Armonk, NY: IBM Corp), two-tailed tests were used, and a *p-*value <0.05 was considered statistically significant.

## Results

**FIGURE 1:**
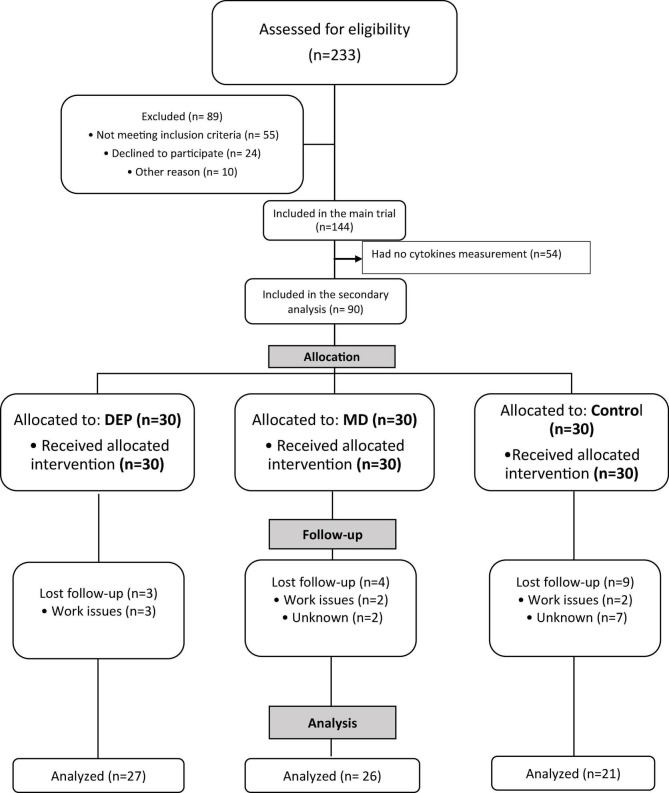
Flowchart of patients included in the study.

A total of 90 women with RA were included in this secondary analysis and were randomly assigned to the three intervention groups (30 patients per group), as shown in Figure 1.

The mean age of all patients was 46.2 ± 11.5 years, with a BMI of 25.8 kg/m^2^. All patients showed low disease activity (DAS28 <3.2). Baseline demographic and clinical characteristics for each study group are presented in Table 1. There were no statistically significant differences in any of the baseline or clinical characteristics, including age, disease duration, comorbidities, disease status, or pharmacological therapy, except for the disability index, with a higher score in the control group compared with the DEP and MD groups. The pharmacologic treatment remained stable during the 24 weeks of the study.

**TABLE 1:** Baseline demographic and clinical characteristics.

Variables	DEP *n* = 30	MD *n* = 30	Control *n* = 30	*p-*value
Age (years)	45.5 ± 11.1	41 ± 10.5	43.3. ± 11	0.86^a^
Weight (Kg)	59.8 (55.4; 66.5)	61.6 (58.7; 75)	61.2 (56.5; 71.8)	0.14^b^
BMI (Kg/m^2^)	25.5 (23.2; 27.3)	25.9 (24.7; 29.2)	26.0 (24.0; 29.3)	0.45^b^
Disease duration (years)	15 (3.8; 20.5)	8 (5.0; 15.0)	9.5 (4.8; 18.5)	0.52^b^
Hypertension n (%)	1 (3.3)	5 (16.6.)	4 (13.3)	0.23^c^
Diabetes n (%)	2 (6.7)	1 (3.3)	2 (6.7)	0.81^c^
Dyslipidemia n (%)	8 (21.6)	9 (22.5)	3 (9.7)	0.58^c^
Hypothyroidism n (%)	6 (20.0)	3 (10)	6 (20)	0.50^c^
Pain (VAS)	6.5 (4.0; 8.0)	5 (2.0; 7.0)	5 (4.0; 7.3)	0.12^b^
DAS28	2.4 ± 1.1	2.5 ± 1.3	2.9 ± 0.9	0.48^a^
CRP mg/dL	0.62 (0.20; 1.34)	0.22 (0.12; 0.50)	0.30 (0.20; 0.70)	0.14^b^
ESR (mm/Hr)	10.5 (5.0; 16.0)	9 (2.8; 16.8)	10 (6.5; 18.0)	0.48^b^
HAQ -DI	0.75 (0.37; 1.25)	0.25 (0; 0.81)	1.0 (0.37; 1.25)	**0.01^b^**
Antimalarials n (%)	11 (29.7)	15 (37.5)	13 (41.9)	0.66^c^
Glucocorticoids n (%)	9 (30)	3 (10.0)	7 (23.3)	0.11^c^
Methotrexate n (%)	20 (66.6)	27 (90)	22 (73.3)	0.12^c^
Sulfasalazine n (%)	4 (13.3)	7 (23.3)	8 (26.6)	0.49^c^
Leflunomide n (%)	7 (23.3)	4 (13.3)	7 (23.3)	0.48^c^

*DEP, Dynamic exercise program; MD, Mediterranean diet; DAS28, Disease Activity Score 28; VAS; Visual analog scale; CRP, C-reactive protein; ESR, Erythrocyte sedimentation rate; HAQ-DI, Health assessment questionnaire disability index. Categorical variables are presented as absolute and relative frequencies and continuous variables are presented as means ± sd or median (25th IQR;75th IQR). Differences between groups were analyzed by ^a^ANOVA or ^b^Kruskal–Wallis, based on ^c^Pearson’s X2 for categorical variables.*

Three of the participants (10%) in the DEP group (*n* = 27), 4 (13.3%) in the MD group (*n* = 26), and 9 (30%) in the control group (*n* = 21) did not complete the follow-up. Reasons included work issues or unknown causes (see Figure 1). Baseline demographic characteristics were similar between patients who completed and those who did not finish the follow-up, as shown in online Supplementary Material.

Before study enrollment, 14 (15.2%) patients reported that they performed physical activity, being this activity more frequent in patients in the DEP group (23.3%), followed by the MD group (20%), and <3% in the control group, without a significant difference between groups (*p* = 0.06). In the DEP group, exercise adherence was 72%.

In the MD group, the MedScore changed from 4.6 to 5.7 (*p* = 0.01), while in the DEP (4 to 4.4) and control groups (4.35 to 3.82) this score did not show a significant change.

Disease activity score-28 did not change, delta in DEP [median (25th IQR;75th IQR)] was −0.05 (−0.9;0.9), MD 0.22 (−0.6;1), control −0.23 (−1.6;0.4) and there were not statistical significant differences between groups (*p* = 0.13).

As shown in Table 2, certain intragroup absolute changes in cytokine concentration occurred 24 weeks after intervention initiation. Thus, TNF-α in the DEP group decreased from 17.8 to 11.6 pg/mL (*p* = 0.04). IL-6 also showed this behavior after the intervention, with a decrease from 4.3 to 2.1 pg/mL (*p* = 0.01) in the MD group. No other significant intragroup changes were found after 24 weeks of intervention.

**TABLE 2:** Cytokine concentrations at baseline and 24 weeks after the intervention.

Variable	DEP *n* = 27	MD *n* = 26	Control *n* = 21
**Pro-inflammatory**			
TNF-α (pg/mL)			
Basal	17.8 (5.6; 36.2)	11.2 (4.2; 17.3)	10.1 (5.8; 20.6)
24 weeks	11.6 (6.0; 27.5)	9.8 (3.2; 12.3)	16.3 (7.2; 29.1)
*p-*value^ d^	**0.04**	0.05	0.28
TNF-β (pg/mL)			
Basal	5.4 (0; 114.2)	2.7 (0; 68.1)	10.1 (5.8; 20.6)
24 weeks	1.8 (0; 46.8)	5.3 (0; 83.0)	17.8 (0; 126.9)
*p-*value^ d^	0.05	0.27	0.22
IL-1β (pg/mL)			
Basal	3.2 (1.4; 14.3)	1.7 (0; 6.4)	3.1 (0.2; 16.5)
24 weeks	3.2 (0.1; 14.7)	3.5 (0.9; – 6.2)	5.8 (0.7; 17.7)
*p-*value^ d^	0.97	0.37	0.54
IL-6 (pg/mL)			
Basal	6.7 (0.6; 13.7)	4.3 (0.4; 9.8)	5.2 (1.6; 20.4)
24 weeks	4.7 (0;16.5)	2.1 (0; 6.2)	7.6 (1.9; 23.7)
*p-*value^ d^	0.90	**0.01**	0.32
**Anti-inflammatory**			
IL-10 (pg/mL)			
Basal	4.9 (0; 13.7)	3.7 (0; 11.1)	6.7 (0; 19.3)
24 weeks	6.0 (0; 11.8)	8.9 (0.6; 19.8)	15.6 (1.8; 51.6)
*p-*value^ d^	0.27	0.87	0.52
IL-1Ra (pg/mL)			
Basal	53.3 (8.7; 196.3)	24.1 (0; 201.9)	40.0 (11.4; 216.2)
24 weeks	31.3 (7.5; 132.8)	89.6 (15.5; 270.9)	79.6 (15.6; 211.3)
*p-*value^ d^	0.09	0.33	0.63

*DEP, Dynamic exercise program; MD, Mediterranean diet; MUFA, Monounsaturated fatty acid; SFA, Saturated fatty acid. Variables are presented as median (25th IQR; 75th IQR).*

*^d^Differences between baseline and final in the groups were using a Wilcoxon matched-pairs test. Bold values indicate p < 0.05.*

**FIGURE 2:**
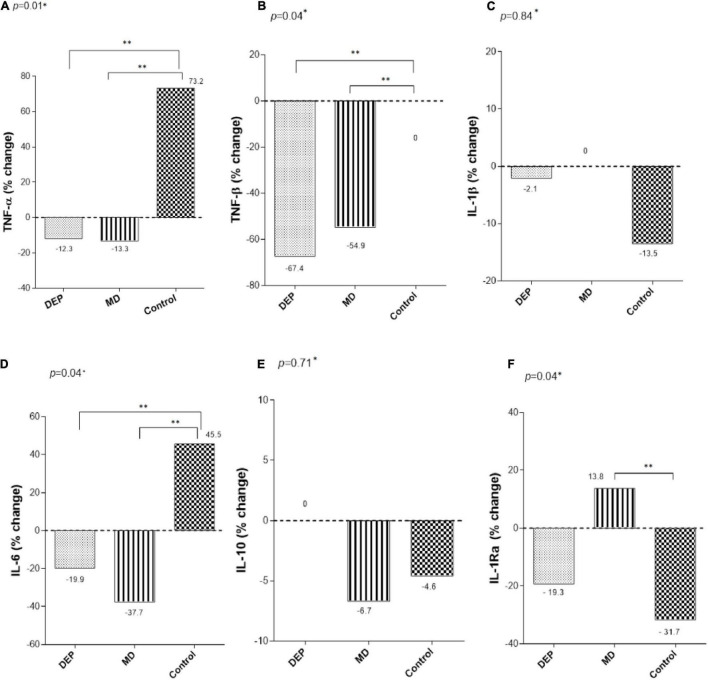
Changes of **(A)** TNF-α, **(B)** TNF-β, **(C)** IL-1β, **(D)** IL-6, **(E)** IL-10, and **(F)** IL-1Ra, after 24 weeks of intervention. *Differences between groups were analyzed by Kruskal-Wallis test. ^**^Post hoc analysis using Mann- Whitney U test with Bonferroni correction *p* < 0.05.

Figure 2 shows the percentage changes in cytokine concentrations after 24 weeks of intervention. TNF-α showed significant decreases in the DEP group (−12.3%) and the MD group (−13.3%), while it increased 73.2% in the control group, with a significant difference among the control and interventions groups (*p* = 0.01). Similarly, TNF-β decreased in the DEP group (−67.4%) and the MD group (−54.9%), while the control group exhibited no changes at the end of the study, with a significant difference among the control and intervention groups (*p* = 0.04). There were, however, no statistically significant differences between DEP and MD groups. On the other hand, the MD group showed a statistically significant difference in IL-1Ra (13.8%) concentration compared to the control group (−31.7%), *p* = 0.04. The other cytokines that were evaluated did not show differences among groups after 24 weeks of intervention. Regarding differences in baseline cytokine concentrations between patients who completed and those who did not finish the follow-up, we observed differences in the cytokines TNF-β and IL-1Ra (see Supplementary Material).

## Discussion

There were some key findings in this secondary analysis investigating the effects of the DEP and MD on serum concentrations of pro-inflammatory and anti-inflammatory cytokines after 6 months of intervention in women with RA. First, implementing a DEP or consuming an MD for 24 weeks presented a statistically significant decreased the serum concentrations of TNF-α, TNF-β, and IL-6 compared with a control group that received only general recommendations concerning diet and physical activity. Second, the MD intervention increased the concentration of IL-1Ra compared to that in the control group and this difference also was statistically significant, suggesting that in RA patients, MD and DEP, implemented as individual interventions, may mediate inflammatory responses.

Immune cells are bioenergetically expensive during activation, which requires tightly regulated control of metabolic pathways. These pathways are essential to the differentiation that immune cells undergo and the resulting cell fates. Thus, these cells are easily affected by the nutritional state and amount of exercise (30). In healthy individuals, exercise exerts an anti-inflammatory effect due to the release of myokines, including IL-6. This release in the acute stage of exercise induces increased production of IL-1Ra and IL-10 by the mononuclear cells of the blood; then, these factors act to decrease the concentrations of pro-inflammatory cytokines such as TNF-α and IL-1β (31–33).

Our results regarding the effects of exercise disagree with those of other studies in similar populations. For instance, a pilot study conducted by Bartlett et al. assessed the effects of 10 weeks of intense exercise in patients with RA and found no significant post intervention changes in the concentrations of IL-6 or TNF-α and, as in our study, found no changes in IL-1β or IL-10 levels (34). On the other hand, Bearne et al. studied the effect of a 5-week rehabilitation program comprising 10 exercise sessions designed to increase quadriceps strength in participants with RA (*n* = 46) and compared them with healthy participants (*n* = 47). No changes were found in any study group in IL-1β, TNF-α, or IL-6 levels (35). The discrepancies between the results found in our study regarding the DEP group and those described above could be attributed to the intensity and type of exercise, the number of participants, and the ethnic groups and characteristics of the patients studied. Furthermore, we did not find significant changes in the concentrations of the anti-inflammatory cytokines (i.e., IL-10 and IL-1Ra) after the implementation of the DEP. We hypothesize that a longer exercise intervention is needed to observe increases in the concentrations of anti-inflammatory cytokines.

The anti-inflammatory effects of the MD are attributed to the considerable amounts of monounsaturated and polyunsaturated fatty acids (MUFAs and PUFAs, respectively) consumed in this diet (36). The current evidence suggests that oleic acid, the main MUFA present in olive oil, does not significantly reduce inflammation in the acute phases of RA. The anti-inflammatory effects of olive oil are evidenced in food patterns consisting of food cooked in olive oil. At the same time, PUFAs are known to be precursors of eicosanoids, which can exert an anti-inflammatory effect by inhibiting cytokines such as TNF-α, IL-1β, IL-6, and IL-8 (37, 38). The effects found for pro-inflammatory cytokines in the MD group were like those reported in previous studies. Mena et al. measured the impact of a MD with olive oil supplements and a MD with nut supplements. They compared them with a control group in a sample of 112 older patients with diabetes or cardiovascular risk factors. The main findings after 3 months of intervention showed that the MD groups exhibited decreased IL-6 concentrations. Concentrations decreased by 17% in the group consuming the MD with olive oil and by 15% in the group consuming an MD with nuts, while concentrations in the control group increased by approximately 22% with a statistically significant difference between groups (*p* < 0.001) (39). Moreover, Torres et al. assessed the effect of a MD in 60 patients with RA and high cardiovascular risk according to the Framingham risk score in comparison with a standard diet, and they reported decreases in the concentrations of TNF-α (2.1 vs. 1.2 pg/mL,*p* < 0.001) and IL-6 (3.8 vs. 2.53 pg/mL, *p* < 0.001) in the MD group (40). Additionally, Chrysohoou et al. described in a large study of healthy adults (*n* = 3042) that those with greater adherence to the MD exhibited concentrations of IL-6 17% (*p* = 0.02) lower than in those with poorer adherence. In contrast, no differences were found in TNF-α concentrations (41). Finally, Esposito et al. conducted a study in 180 metabolic syndrome patients, and their results suggested that implementing a MD for 2 years lowers the concentrations of IL-6, IL-7, and IL-8 (42). However, the evidence for the effect of the MD on inflammation in patients with RA is controversial, so appropriately designed clinical trials are needed to support our results, since studies measuring inflammatory markers are components of secondary analyses and are not conclusive (43).

It is important to note that the term immunometabolism has been used recently, since the immune system monitors and responds to specific metabolic changes and is very sensitive to environmental changes to maintain systemic homeostasis. Diet and physical fitness status are the main environmental factors that affect the function of the immune system. An excess or deficiency in nutrients alters the immune response; therefore, the inclusion of the metabolic profile and the physical status, as well as the interaction between these factors, is essential in studies where inflammatory markers are evaluated (44, 45).

We found differences in the baseline cytokine concentrations of the participants who completed the follow-up and those who abandoned the study (see Supplementary Material); this is important to mention since the subjects who completed the follow-up had higher concentrations of cytokines, so our results are probably only replicable in participants with RA who exhibit high concentrations of cytokines. Also, differences in the baseline disability index between groups could lead to potential bias in the results. This could explain why the control group had a smaller percentage of patients who did physical activity at the start of the research, since these patients had a higher baseline disability score.

The present study has some limitations. First, our results are not conclusive because it is a secondary analysis, so further research is needed with larger sample sizes and robust methodologies. Second, the loss of participants in the control group exceeded 10%, which could have affected the results. Third, the metabolic profile (i.e., glucose, lipoproteins, and insulin levels) was not assessed, and therefore, it was difficult to understand the inflammatory profile of the participants. Fourth, there is limited generalizability of the results given that only women were included and, more importantly, a group of healthy women was not entered as a control to have a comparison value of the anti- and pro-inflammatory cytokines studied. Unfortunately, at present, there are no standardized reference values for these molecules. Fifth, some of the results are based on the estimation of diet based on a questionnaire. Finally, the pharmacologic treatment effect on the cytokine profile was not assessed, as a control group without pharmacologic treatment was not evaluated.

## Conclusion

The outcome of the present study suggests that women with RA with low disease activity after 24-week intervention of a DEP or an MD may decrease the percentage change of serum concentrations of pro-inflammatory cytokines (TNF-α, TNF-β, IL-6) compared to the control group. In contrast, the consumption of an MD increases the percentage change of serum concentrations of the anti-inflammatory cytokine IL-1Ra compared to the control group.

## Data Availability Statement

The raw data supporting the conclusions of this article will be made available by the authors, without undue reservation.

## Ethics Statement

The studies involving human participants were reviewed and approved by Instituto Nacional de Ciencias Médicas y Nutrición SZ Human Ethics and Research Committees. The patients/participants provided their written informed consent to participate in this study.

## Author Contributions

JP-J, LC-M, JA-V, and LL designed the study. ML-M, LC-M, and MO-M analyzed data and wrote the manuscript. AH-A performed clinical evaluation of the patients. MO-M and JG-M implemented nutritional and exercise protocols. ML-M and MO-M collected the data. AH-A and LL edited the manuscript. All authors have reviewed the manuscript and approved its submission.

## Conflict of Interest

The authors declare that the research was conducted in the absence of any commercial or financial relationships that could be construed as a potential conflict of interest.

## Publisher’s Note

All claims expressed in this article are solely those of the authors and do not necessarily represent those of their affiliated organizations, or those of the publisher, the editors and the reviewers. Any product that may be evaluated in this article, or claim that may be made by its manufacturer, is not guaranteed or endorsed by the publisher.
