# Supplementary material for: Comparison of the Impacts of a Dynamic Exercise Program vs. a Mediterranean Diet on Serum Cytokine Concentrations in Women With Rheumatoid Arthritis. A Secondary Analysis of a Randomized Clinical Trial
Source: Front Nutr. 2022 Apr 25;9:834824. doi: 10.3389/fnut.2022.834824 (PMC9082589; doi:10.3389/fnut.2022.834824)
Supplement: Supplementary file 1 [file Data_Sheet_1.docx]

**Supplementary Material 1. Nutrients targets and average daily servings of foods in the Mediterranean diet group**

| Nutrients | (% of total kcal) |
| --- | --- |
| Carbohydrates | 50 |
| Fats | 30 |
| Proteins | 20 |
| Food group |  |
| Whole grains (no. servings/meal) | 1-2 |
| Fresh fruits (no. servings/day) | 2-4 |
| Vegetables (no. servings/meal) | 2-3 |
| Fish seafood (no. servings/wk) | > 2 |
| Tree nuts and peanuts (no. servings/day) | 1-2 |
| Legumes (servings/wk) | > 2 |
| Olive or canola oil (no. servings/day) | 5 |
| Red meat (servings/wk) | < 2 |

| **Supplementary Material 2. Summary of the prescribed dynamic exercise program** | | | | | |
| --- | --- | --- | --- | --- | --- |
| **Time (Frequency)** | **Exercise** | **Type** | **Mode** | **Repetion / Sets** | **Time(min)** |
| **Outpatient exercise intervention (adaptative phase) 2 Weeks / twice a week – 80 min per day** | ROM exercise | Active | Static | 10 x 1 | NA |
|  | Aerobic | Initially at low intensity 45 - 55 % HR | Treadmill | NA | 15 min |
|  | Resistance training | Against Gravity | Functional Movements patterns. | 10 x 1 | NA |
|  |  | (isometric and isotonic upper and lower extremities large muscular groups settings) |  |  |  |
|  | Cool down | Static Stretching – all muscle groups worked at session. | Static Stretching | NA | 10 min |
| **Outpatient exercise intervention First 3 months 12 Weeks / twice a week – 80 min per day** | ROM exercise | Active | Static | 10 x 2 | NA |
|  | Aerobic | Low - Moderate intensity 55 - 65% HR | Treadmill – Static Bike | NA | 25 min |
|  | Resistance training | Against Gravity – Light weight | •    Functional movement’s patterns. Multi-joint or single – joint exercises. | 10 x 2 | NA |
|  |  | (isotonic large muscle groups settings) | •    Wooden Sticks (< 1kg) |  |  |
|  |  | 50 % - 1RM per repetition | •    Elastic bands (1,5 - 2,5 kg) |  |  |
|  |  |  | •        Resistance was gradually increased |  |  |
|  |  |  |  |  |  |
|  | Recreational game-session | Games base on Sport exercises | •    Multi-joint exercises | 8 -10 x 2 | 20 min |
|  |  | •        Gestures from basketball, football and volleyball. | •    Air Plastic balls (no weight) |  |  |
|  |  | Integrating flexibility, strength and coordination exercises. | •    Circuits |  |  |
|  |  |  | o 5 – 7 stations |  |  |
|  |  |  | o Difficulty was gradually increased every 2 weeks |  |  |
|  | Cool down | Static Stretching – all muscular groups worked at session. | Static Stretching | Na | 10 min |
| **Outpatient exercise intervention Last 3 months 12 Weeks / twice a week – 80 min per day** | ROM Exercise | Active | Static | 15 x 2 | NA |
|  | Aerobic | Moderate intensity 65% - 75 % HR | Treadmill – Static Bike | NA | 30 Min |
|  | Resistance Training | Against Gravity – Light weight (isotonic large muscular groups settings) | •    Functional Movements patterns. Multi-joint or single – joint exercises. | 15 x 2 | NA |
|  |  | 65% - 1RM per repetition | •    Wooden Sticks (< 1kg) |  |  |
|  |  |  | •    Elastic bands (2,5 - 3,5 kg) |  |  |
|  |  |  | •        Resistance was gradually increased |  |  |
|  |  |  |  |  |  |
|  | Recreational game-session | Games base on Sport exercises | •    Multi-joint exercises | 10 -15 x 2 | NA |
|  |  | •        Gestures from basketball, football and volleyball. | •    Air Plastic balls (no weight) |  |  |
|  |  | Integrating flexibility, strength and coordination exercises. | •    Circuits |  |  |
|  |  |  | o 4 – 6 stations |  |  |
|  |  |  | o Difficulty was gradually increased every 2 weeks |  |  |
|  | Cool down | Static Stretching – all muscle groups worked at session. | Static Stretching | NA | 15 min |
| ROM, Range of motion exercise; HR, Heart rate; RM, Repetition maximum. | | | | | |

**Supplementary Material 3. Baseline demographic, clinical characteristics and cytokine profile after follow-up in patients that completed the study or lost follow-up.**

| Variable | | Completed follow-up  n=74 | | Lost to follow-up  n= 16 | | *p* value |
| --- | --- | --- | --- | --- | --- | --- |
| Age (years) | | 46.4 ± 11.2 | | 45.7 ± 12.7 | | 0.79 |
| Weight (Kg) | | 60.1 (56.3 – 70.8) | | 61.6 (58 – 72) | | 0.58 |
| BMI (Kg^2^/m) | | 25.7 (23.4 – 29.1) | | 25.6 (24.1 – 28.6) | | 0.99 |
| Disease duration (years) | | 11 (4 - 19) | | 8 (7 – 16) | | 0.70 |
| Pain (VAS) | | 6 (4 – 8) | | 5 (3 – 8) | | 0.73 |
| DAS28 | | 2.6 ± 1.1 | | 2.8 ± 1.4 | | 0.66 |
| CRP mg/dL | | 0.37 (0.17 – 0.86) | | 0.29 (0.19 – 0.47) | | 0.68 |
| ESR (mm/hr) | | 9.5 (4.3 - 16) | | 11 (6 – 23) | | 0.46 |
| HAQ-DI | | 0.62 (0.12 – 1.15) | | 0.75 (0.25 – 1.25) | | 0.61 |
| Cytokines | | | | | | |
| Pro-inflammatory |  | |  | |  | |
| TNF-α | | 9.8 (5.6 – 17.3) | | 11.6 (4.4 – 19.1) | | 0.58 |
| TNF-β | | 15.4 (0.3 – 115.9) | | 0 (0 – 16.8) | | 0.003 |
| IL-1β | | 2.8 (0.6 – 15.7) | | 1.6 (0.3 – 7.6) | | 0.24 |
| IL-6 | | 5.4 (1.3 – 13.3) | | 5.1 (0.4 – 21) | | 0.72 |
| Anti-inflammatory | |  | |  | |  |
| IL-10 | | 5.4 (0 – 18) | | 1.32 (0 – 9.3) | | 0.09 |
| IL-1Rα | | 62.2 (15.4 – 235.8) | | 26 (0 – 69.9) | | 0.005 |

BMI, Body mass index; DAS28, Disease Activity Score 28; VAS, visual analogue scale; CRP, C-reactive protein; ESR,

erythrocyte sedimentation rate; HAQ-DI, Health Assessment Questionnaire

Categorical variables are presented as absolute and relative frequencies and continuous variables are presented as means± standard deviation.

or median (p25 - p75).
